# Establishing best practices guidelines for collaborative research in global surgery: developing a Delphi survey

**DOI:** 10.7189/jogh.15.04237

**Published:** 2025-09-05

**Authors:** Ramitha Eshan Ruwanpathirana, Swati Deshpande, Sophia Abdulhai, Mathilde Djeneba Billau, Sumeja Catic, Theresa L Chin, Julia N Harrison, Pratyush Kumar, Maria Cecilia T Leyson, Helen W Li, Katayoun S Madani, Arthur Serumaga, Margaret Tarpley, Grayson Wright, Nia N Zalamea, Joshua S Ng-Kamstra

**Affiliations:** 1 Manipal College of Medical Sciences, Pokhara, Nepal; 2 Micaehl E DeBakey Department of Surgery, Center for Global Surgery, Baylor College of Medicine, Houston, Texas, USA; 3 Association of Academic Global Surgery, Milwaukee, Wisconsin, USA; 4 Jawaharlal Nehru Medical College, Datta Meghe Institute of Higher Education and Research, Department of General Surgery, Wardha, India; 5 Department of Surgery, Indiana University School of Medicine, Indianapolis, Indiana, USA; 6 Medical Faculty of the University of Sherbrooke, Sherbrooke, Quebec, Canada; 7 Branch for Global Surgical Care, Faculty of Medicine, University of British Columbia, Vancouver, British Columbia, Canada; 8 Department of Family Medicine, JU ‘Dom zdravlja’ Zenica, Zenica, Bosnia and Herzegovina; 9 University of California Irvine, Department of Surgery, Irvine, California, USA; 10 Department of Surgery, University of Minnesota, Minneapolis & St Paul, Minnesota, USA; 11 Dr. Baba Saheb Ambedkar Medical College and Hospital, Rohini, India; 12 Department of Surgery, Ospital ng Maynila Medical Center, Manila, Philippines; 13 Department of Surgery, Washington University, St Louis, Missouri, USA; 14 Department of Surgery, Kayunga Regional Referral Hospital, Kayunga, Uganda; 15 Department of Surgery, Vanderbilt University, Nashville, Tennessee, USA; 16 Department of Medical Education, University of Botswana, Gaborone, Botswana; 17 University of Tennessee Health Science Center, UTHSC Global Surgery Institute, Memphis, Tennessee, USA; 18 Department of Surgery, Mass General Brigham, Boston, Massachusetts, USA; 19 Department of Global Health and Social Medicine, Harvard Medical School, Boston, Massachusetts, USA

## Abstract

**Background:**

Collaborative research in global surgery has resulted in the rapid development of the field via knowledge creation and dissemination, research capacity building, and direct improvements in the delivery of clinical care. Yet the establishment and maintenance of trans-boundary collaborations carries significant risk to health systems, clinicians, patients, and researchers, particularly if such collaborations are not developed thoughtfully and with appropriate guardrails. In recent years, there has been significant growth in the literature on the pervasiveness and impact of neocolonialism on global health research.

**Methods:**

To harness the benefits of global surgery research collaboration while mitigating these risks, we reviewed the literature, collaboratively developed best practice statements, and iteratively refined these.

**Results:**

Eighty-one best practice statements were developed across six themes: establishing partnerships, managing power imbalances, addressing equity in funding, ensuring high ethical standards in Human Subjects Research, maintaining quality and rigor in scientific work, and long-term planning.

**Conclusions:**

These statements will form the basis of a Delphi process to establish a robust set of guidelines for best practice in collaborative global surgery research.

Global surgery, an interdisciplinary field dedicated to promoting equity and improving outcomes in surgical care, has achieved recognition as a critical component of global health [1]. Collaborative research models, often involving individuals and institutions from multiple countries, are commonly used to address knowledge gaps and improve care. However, power imbalances, inequitable funding structures, and neocolonial approaches that run counter to the equity imperative in such partnerships can stymie progress and cause harm to vulnerable parties and ultimately surgical systems. To maximise benefit and minimise harm, best practice guidelines that ensure an equity-focused approach, methodological rigor, and high ethical standards are needed [2,3].

Resources have been developed for fostering sustainable global health partnerships [3], but these have had limited uptake among surgical collaborations. Moreover, there has been an immense body of literature published in the last several years addressing anticolonial approaches in global health. We aimed to translate this literature into practice, distilling its important insights into a series of statements comprising a Delphi survey [4]. We sought to create an inclusive and systematic process to harness the power of the published literature and the expertise and experience of diverse stakeholders to reach a consensus on the crucial principles and practices necessary for equity in surgical research partnerships. In this paper, we describe our process, key findings from the literature including a set of 81 statements, and our proposed next steps in creating a set of meaningful best practice guidelines.

## METHODS

The establishment of the authorship group began with self-selection of interested individuals from the Research and Ethics Committees of the Association for Academic Global Surgery. To establish reasonable geographic and gender representation within a small group of committed individuals, these members invited representatives of collaborating organisations and active research partners. The group ultimately consisted of 11 female and five male co-authors with current institutional affiliations in Canada, Bosnia and Herzegovina, Botswana, India, Nepal, The Philippines, Uganda, and the USA. Including the national origin of authors, all WHO regions are represented.

We first conducted an asynchronous open-ended survey of the group to agree on key issues in global surgery partnerships. Through this process, we identified six themes: challenges in establishing partnerships, managing power imbalances, addressing equity in funding, ensuring high ethical standards in Human Subjects Research, maintaining quality and rigor in scientific work, and long-term planning. Teams were formed to review the literature on each of these subjects. Each team involved at least one individual from a low- or middle-income country (LMIC). The search process was tailored to each domain, using a purposive and iterative process that drew on seminal works, snowball techniques, traditional database searches, and Artificial Intelligence (AI)-based search and analysis methods (Elicit [5]) to ensure that the most relevant literature was included. Teams summarised the individual themes and created a list of recommendation statements.

After compiling statements from each team, we met virtually as a full authorship group to review and refine these statements. We scheduled meetings to accommodate all time zones and encouraged active participation from all authors. All authors had access to a shared cloud-based document which we used to collaboratively edit statements in real-time. To ensure that all perspectives were represented, we continued discussion until we achieved consensus, requiring both a primary vote and a second to close discussion on each statement. Once all statements were reviewed, we again reviewed the whole body of statements to minimise duplication and ensure internal consistency.

## RESULTS

### Establishing partnerships: components of an equity-oriented memorandum of understanding

During the formation of a research partnership, partners can draft an equity-based memorandum of understanding (MoU) setting the terms of the partnership and committing the partners to maintaining an equitable relationship based on clearly defined rights, trust, and transparency [3]. Such an MoU can also provide critical operational guidance from the outset.

The literature provides frameworks and tools for building partnerships and expounds on principles that should be addressed in agreements between partners. Three notable publications provide a set of foundational concepts in partnership building: Global Health Partnerships (formerly Tropical Health and Education Trust, THET) Guidance for New Health Partnerships including the organisation’s Principles of Partnership [6], a 2016 qualitative study on principles for successful research partnerships in global health [7], and a 2023 pragmatic paper on equitable global health partnerships in academic health sciences [8]. Other guidelines and tools are described (**Table 1**). Note that prior guidelines-focused work done involving the Association of Academic Global Surgery (AAGS) was not specific to research collaborations [25].

**Table 1:** Guidelines, frameworks, principles, tools, and codes of conduct for forming equitable global health research partnerships

Title of publication	Authors	Publication year	Type
Moving to research partnerships in developing countries [9]	Costello & Zumla	2000	Checklist
North-South Research Partnerships: Issues and Challenges [10]	Netherlands Development Assistance Council (RAWOO)	2001	Principles
Partnership Assessment Toolkit (PAT) [11]	Afsana, et al. (Canadian Coalition for Global Health Research, CCGHR)	2009	Toolkit
The CCGHR Principles for Global Health Research: Centering equity in research, knowledge translation, and practice [12]	Plamondon & Bisung (CCGHR)	2015	Principles
Guide to Constructing Effective Partnerships [13]	ELRHA	2012	Guideline
Council on Health Research for Development (COHRED) Fairness Index for international collaborative partnerships [14]	Musolino, et al. (COHRED)	2013	Standard
Research Fairness Initiative (RFI) Summary Guide [15]	Council on Health Research for Development (COHRED)	2018	Guideline
The Partnerships Analysis Tool [16]	Victoria Health Promotion Foundation	2016	Toolkit
Developing a framework for successful research partnerships in global health [7]	Larkan, et al.	2016	Framework
Partnerships for Global Child Health [17]	Steenhoff, et al.	2017	Principles
Rethinking research partnerships: Discussion guide and toolkit [18]	Cornish, et al.	2017	Toolkit
Guide for Transboundary Research Partnerships: 11 Principles and 7 Questions [19]	Swiss Commission for Research Partnerships with Developing Countries (KPFE)	2018	Guideline
Principles for Fair and Equitable Research Collaborations [20]	Rethinking Research Collaborative	2018	Principles
The TRUST Code: A Global Code of Conduct for Equitable Research Partnerships [21]	TRUST	2018	Code of Conduct
Tropical Health and Education Trust (THET) Principles of Partnership [6]	THET	2020	Principles
Sustaining Technical and Analytic Resources (STAR) Partnership Assessment Toolkit [22]	STAR	2021	Toolkit
Global Health Partnerships and the Brocher Declaration: Principles for Ethical Short-Term Engagements in Global Health [23]	Prasad, et al.	2022	Principles
The Equity Tool for Valuing Global Health Partnerships [24]	Larson, et al.	2022	Toolkit
Striving towards true equity in global health: A checklist for bilateral research partnerships [3]	Hodson, et al.	2023	Checklist
Cultural competency and ethical behavior for collaboration in limited-resource settings: Guidelines from the Society of University Surgeons Academic Global Surgery Committee and the Association for Academic Global Surgery [25]	Yang, et al.	2024	Guideline

Across the literature, a set of clear guiding principles emerges:

1. Mutual trust and respect

2. Equitable power sharing and inclusion

3. Mitigation of discrimination

4. Equitable sharing of benefits, responsibilities, resources, and data

5. Effective communication utilising inclusive language

6. Fair acknowledgement of contributions

7. Sustainable capacity building

8. Ethical conduct of research

9. Effective monitoring and evaluation

10. Fair conflict resolution

11. Transparency and accountability

12. Planning an exit strategy

These principles are reflected in a set of 16 recommendation statements (**Table 2**). Embedding these components into a MoU encourages the collaborators of a research partnership to operate in a mutually beneficial manner where power is distributed fairly and the voices of all stakeholders are heard and valued, especially those from historically marginalised groups and individuals from resource-constrained settings.

**Table 2:** Collaboratively developed best practice statements for Delphi survey on the theme of establishing partnerships

1	Establishing partnerships: components of equity-oriented memoranda of understanding
1.1	Partnerships should be established and maintained based on mutual trust, credibility, authenticity, shared strategic objectives, mutual responsibility, collaborative authority, and collective outcomes [24,26,27].
1.2	Collaborating partners should cultivate respect for the histories, cultures, needs, capabilities, opinions, and contexts of the involved communities [3,23,24,26,28,29].
1.3	Visiting partners should acknowledge their limited perspectives as non-members of host communities [3,23].
1.4	Memoranda of understanding should be co-developed by all stakeholders, prioritizing the interests, needs and culture of LMIC partners.
1.5	Memoranda of understanding should include an a priori commitment to offer educational and research opportunities to lower-income country team members to mitigate inequities of opportunity in partnerships between countries of different income levels [3,7,27].
1.6	Memoranda of understanding should establish clear roles and responsibilities among collaborating partners [30-32].
1.7	Memoranda of understanding should establish fair access to project samples, data, and results, and guidelines for their usage in other manuscripts and/or projects, in compliance with local laws governing data use [3,7].
1.8	Memoranda of understanding should establish fair access to data analysis and dissemination resources (*e.g*. access to consultants), as well as other relevant technological tools (*e.g*. statistical and word processing software) [3,7].
1.9	Memoranda of understanding should establish criteria for authorship and acknowledgements based on the individual contributions of team members [3,7].
1.10	Memoranda of understanding should establish recognition and notification protocols to ensure that all team members are acknowledged when their work is utilized in other manuscripts and projects [3,7].
1.11	Memoranda of understanding should include a commitment to the collaborative development of a Monitoring, Evaluation, and Learning framework to define the main goal and assess its attainment by specifying the objectives, outcomes, activities, and indicators [8,33].
1.12	Memoranda of understanding should include a commitment to regular risk assessments to mitigate unforeseen changes, impacts, and risks [24,26].
1.13	Memoranda of understanding should reference processes to manage conflicts or disagreements between collaborating partners to ensure fairness and equity [24,26].
1.14	Memoranda of understanding should reference transparent decision-making processes, especially in governance, finances, operations, and performance [24,26,30,32].
1.15	Memoranda of understanding should outline how the likely benefits of the partnership, including any monetary or intellectual property outputs, will be shared.
1.16	Memoranda of understanding should include a commitment to exit interviews between partner organizations at the conclusion of any project to ensure that goals are met [7,32,34].

### Strategies to mitigate power imbalances in research collaborations.

Power imbalances develop when decision-making authority is either tacitly or explicitly dominated by one partner (or group of partners), to the detriment of others or to the goals of the partnership [35]. The COVID-19 pandemic exposed the sharp power divides in global health: wealthy nations controlled the resources necessary to protect populations from harm, leading to a highly inequitable vaccine rollout and the preventable loss of many lives [35]. Understanding the types of power and the ways that they impact partnerships can ensure that global surgery partnerships are beneficial rather than harmful [36].

Taxonomies of power have included such categories as epistemic (derived from ‘expertise’ or knowledge-based claims) [37], normative (derived from claims of moral authority) [38], compulsory (involving direct control of one group or another) [39], institutional (indirect power wielded via control of institutions) [39,40], structural (involving rules, regulations, and the social arrangement of actors) [41], and productive power (referring to the ability to produce meaning, discourse, and identity) [41]. Using another lens, power imbalances in global health are rooted in colonialism and racism [35].

Pragmatically, it is critical to note that power dynamics are inherent in every aspect of global health [38], and at every level from individual to international. Thus, discussions of power are relevant to all research partnerships, regardless of the scale of the partnership or income level of the participating parties.

Power imbalances in surgical research collaborations can result in such inequities as inequality of opportunity (*e.g*. a few ‘well known’ LMIC individuals are selected as perennial research partners of high income country (HIC) groups), relegation of LMIC partners to the conduct of technical rather than intellectual work, and dominant contributions from individuals with ‘protected research time’ (a rarity in the Global South). Direct harms can also result, including research waste (*e.g*. a research agenda that is not locally relevant); disruption of surgical care to meet the clinical or research needs of international partners, and intellectual theft (failing to recognise the academic contributions of partners).

Efforts to mitigate power imbalances must be ongoing processes rather than one-time activities. Based on the existing literature, we have developed 12 statements to assist research partnerships in this work (**Table 3**). These statements address several key activities including the meaningful integration of diversity, equity, and inclusion work into the partnership, practical aspects of partnership work (including times and locations of meetings, workflows, and funding flows), the importance of multilevel partnerships (including individuals at all levels of training), and the unique role of South to South collaborations (partnerships between institutions with shared histories and geographies) in avoiding the pitfalls inherent in HIC-LMIC partnerships.

**Table 3:** Statements on the theme of power imbalances

2	Power imbalances: strategies to minimize the dominance of powerful voices
2.1	Representatives of a collaborative partnership should jointly acknowledge and address how the various sources of power held by members (including financial, institutional, academic, historical/colonial, and normative power) could impact their working relationship [35-38].
2.2	Representatives of a collaborative partnership should jointly acknowledge and address how racism could impact their working relationship and examine for biases, assumptions, beliefs, and motivations rooted in racism [39]. N.B. To evaluate this statement consider this definition of racism as used by the American Public Health Association: ‘Racism is a system of structuring opportunity and assigning value based on the social interpretation of how one looks (which is what we call ‘race’), that: ● unfairly disadvantages some individuals and communities, ● unfairly advantages other individuals and communities, and ● saps the strength of the whole society through the waste of human resources.’ [42]
2.3	Representatives of a collaborative partnership should jointly acknowledge and address the ongoing impact of colonialism on their working relationship and examine for biases, assumptions, beliefs, and motivations rooted in colonialism [39]. N.B. To evaluate this statement consider this definition of colonialism: ‘We define colonialism as the state-sponsored construction of non-merit inequality for the benefit of one group at the expense of another. These non-merit inequalities circumscribe practically every aspect of global health, including in geography, knowledge, language, prominent journals, analytic methods, institutions, and categories of individual and group identity such as race, gender, ethnicity, and lived experience.’ [43]
2.4	All partnership members should jointly acknowledge that power dynamics are inherent to collaborative relationships and mitigating their deleterious effects requires continuous effort throughout the life of the partnership.
2.5	Partnership members should use a formal partnership assessment framework/tool to promote effectiveness, sustainability, and equity, and mitigate the impacts of power imbalances [40].
2.6	Diversity, equity, and inclusion should be integrated into governance structures on each side of the partnership, including marginalized perspectives in decision-making processes [39].
2.7	Partnerships should work to acknowledge, respect and transparently share agendas, conflicts of interest, and divergent interests held by each member.
2.8	Governance structures within the partnership should be established and documented a priori with agreement from all parties, with clear decision-making roles and processes for consensus, disagreement, and conflict management [41].
2.9	Leadership and governance structures should continually be evaluated with the goal of redistributing power across all partners to ensure shared design, shared implementation, and shared credit/merit.
2.10	Partnership members should jointly acknowledge and address how practical aspects of the working relationship, such as times and locations of meetings, workflows, and funding flows are influenced by power imbalances.
2.11	To promote power-sharing at all levels of research or clinical training, partnerships can consider operating under a working model in which partners are matched with counterparts at respective levels of training [36].
2.12	To minimize the impacts of power imbalances, relationships between partnering organizations with shared histories and geographies (eg, ‘Southern Networks’) should be promoted, decentralizing institutions and communities with historical and financial power [35,39].

### Funding collaborative research: equity considerations for funding agencies and applicants

Funding organisations and mechanisms play a pivotal role in fostering equitable research partnerships. The structure of funding opportunities and how they are adjudicated can incentivise a priori attention to common equity concerns. Furthermore, funders can hold researchers accountable for collaboration practices prior to distribution of funds [44,45]. In parallel, researchers can seek funding opportunities that promote equity rather than entrench power imbalances. In reviewing the relevant research, we drafted 14 statements that address the role of both funders and applicants in promoting equity (**Table 4**).

**Table 4:** Statements on the theme of funding collaborative research

3	Funding collaborative research: equity considerations for funding agencies and applicants
3.1	Funders should enforce conditions holding researchers accountable for equitable collaboration and equitable distribution of research incentives and rewards [44,45].
3.2	Funders should allocate funds prioritizing projects addressing population needs as determined by local clinicians, researchers, and the populations they serve [46].
3.3	Funders should promote equity in resource allocation by establishing clear funding principles with explicit requirements to address barriers faced by LMIC research collaborators [3,46], reimbursing indirect research costs, and expanding reimbursement for activities traditionally limited to HICs [47].
3.4	Transparent funding with minimal conflicts of interest should be ensured through stringent measures. These include mandating the declaration of all funding sources, keeping investors separate from the funding process, reporting clear selection criteria for applicants, providing clear communication channels, and offering timely feedback and progress updates to applicants [48].
3.5	Funders should promote research capacity-building by creating funding opportunities that require a primary investigator from the country in which the research is conducted [44,49].
3.6	Funders should minimize barriers to grant applications by allowing applications in languages other than English, providing application support and user-friendly application platforms, and using short proposal screens to minimize time spent on unfunded applications [47,48,50].
3.7	Funding panels should be demographically and geographic diverse and should incorporate layperson and researcher perspectives from the regions and populations in which the research will be conducted, via early proactive consultation [3,46,48,51].
3.8	Applicants for grants to fund collaborative work should sign an equity-oriented memorandum of understanding referencing resource and incentive allocation processes that are in keeping with the terms and conditions of the funding agency.
3.9	Collaborative grant proposals should include clearly defined goals, measurable and achievable targets, feasible timelines, and a reasonable budget [3,46,50-52].
3.10	Collaborative grant applicants should identify funding opportunities that align with their common research goals, address the needs of the local population, and are flexible enough to respond to changing local priorities [50,52].
3.11	Partnerships should look to increase direct funding to LMIC partners.
3.12	In partnerships where care is delivered directly, partners should commit to delivering care regardless of patients’ ability to pay.
3.13	Funding for partnerships should come from diversified sources and can include not-for-profit entities, for-profit entities, direct revenue streams, private support and institutional support.
3.14	Financial resources should be equally controlled by LMIC and HIC partners.

Funders can employ several strategies to reduce barriers to funding for researchers from low- or middle-income countries. First, they can create funding opportunities that require a primary investigator from the country where the research is conducted. By investing in local research leadership and expertise, funders can empower LMICs to take ownership of their research agendas and drive sustainable development within their communities [48,50]. Second, funders create a more inclusive, diverse, and supportive research ecosystem by streamlining application processes and reducing administrative burdens. Barriers can be further lowered by allowing applications in languages other than English, providing application support, and utilising user-friendly application platforms [44,47,48,51]. To reduce power differentials, funders can require that financial resources be at least equally controlled by LMIC and HIC partners, with direct funding to LMIC institutions where possible. Finally, funders can facilitate the development of solutions that are contextually relevant and directly benefit the communities in which they are implemented by ensuring that funding priorities are informed by clinicians, researchers, and the communities they serve [3,47].

Applicants should seek funding opportunities that align with their common research goals, address the needs of the local population, and are flexible enough to respond to changing local priorities [50,52]. Application-specific memoranda of understanding should reference resource and incentive allocation processes consistent with the terms and conditions of the funding agency. Collaborative grant proposals must include clearly defined goals, measurable and achievable targets, feasible timelines, and a reasonable budget [3,46,50-52]. Finally, to promote stability in cash flows, funding for partnerships should come from diversified sources and can include not-for-profit entities, for-profit entities, direct revenue streams, private support and institutional support.

### Human subjects research: consent, protection, and oversight

Human subjects research must follow the highest ethical standards laid out in landmark publications such as the Nuremberg Code [53], Belmont Report [54], Council for International Organizations of Medical Science (CIOMS) [55] and International Council for Harmonization of Technical Requirements for Pharmaceuticals for Human Use Guideline for Good Clinical Practice (ICH- GCP) guidelines [56]. Collaborative, multinational, global surgery research poses difficulties due to varied cultural, sociopolitical, economic and linguistic differences between sites. The technological and logistical setting of one collaborating institution may differ significantly from another, creating pitfalls for the ethical conduct of research. The existing literature has described the application of ethical principles across every step in the research process.

As is discussed in the next section, collaborative agenda-setting is critical. Next, research planning and implementation should be done thoughtfully, under the oversight of local or international Institutional Review Boards (IRBs) [57]. Investigators should establish clear protocols that minimise bias in participant recruitment, use consent practices that are robust and well-understood by all participants, and that integrate routine monitoring and reporting practices and timely audits [57-60].

In the internet age, data security and the confidentiality of participants are of paramount importance, and protocols should reflect this. Recent literature has emphasised the importance of local data ownership and storage, and of sharing results with the community prior to publication [61]. The sharing of bio-specimens and digital imaging can be done ethically, but this requires a priori consent or a dynamic consent process to prevent unethical secondary use, and an understanding that primary ownership of the specimens is held by the communities from which they are derived [62–64].

Finally, the ethics of authorship has been extensively described in the literature. Early in the research process, discussions regarding authorship are essential, ensuring that all contributors are appropriately recognised. First- and last-authorships are important in most academic environments, and so the relegation of LMIC partners to middle author positions is not a substitute for true engagement that merits key author positions [65].

Organising international collaborative research projects with high-income and low-income partners requires conscious and intentional efforts to protect at-risk populations and reduce power inequities. To maximise the benefits of partnering without abusing or harming local participants, ethical norms must be applied carefully throughout the study process. We have drafted 11 statements addressing these principles (**Table 5**).

**Table 5:** Statements on the theme of Human Subjects Research

4	Human Subjects Research: consent, protection, and oversight
4.1	The risks and benefits of collaborative global surgery research should be assessed by all partners continuously throughout the research study process, including planning, implementing, analyzing research, and disseminating its knowledge.
4.2	The physical, mental, emotional, and financial impact of collaborative research should be discussed and any harms, which can be unintentional, should be minimized wherever possible [59,60].
4.3	Recruitment of human subjects should be autonomous, beneficial to participants with no or minimal harm, and judicious including all diverse ethnic, socioeconomic, cultural, and linguistic groups with added protection for vulnerable populations.
4.4	All global ethical guidelines for human subjects research such as the Nuremberg Code [53], the Belmont Report [54], Council for International Organizations of Medical Sciences (CIOMS) [55], and International Council for Harmonization (ICH) – Good Clinical Practice (GCP) [56] guidelines along with local ethical guidelines should be followed to ensure the highest level of ethical research.
4.5	All research personnel from both HICs and LMICs should have up to date human subjects training to ensure the highest ethical standards.
4.6	Informed and documented consent should consider cultural, sociopolitical, and linguistic issues with support from local institutional/national ethical committees to ensure the human subjects are as informed as possible [62,63].
4.7	More than one ethics or IRB approval may be necessary to ensure the highest standard of ethics is maintained in all international research [64]. International IRBs / national IRBs for multisite/multi-national collaborative research would help to standardize the process.
4.8	In research projects involving bio-specimens/ digital imaging, consent for use should be obtained with the consent for the study unless a dynamic consent can be obtained to allow for autonomy and prevent their unethical secondary use [65].
4.9	Data safety monitoring boards (DSMB) should be in place for all collaborative research particularly ones involving online data transfers to ensure privacy and confidentiality are not breached for the participants. The DSMB should also monitor for ethical issues or power imbalances for the global surgery research.
4.10	Authorship for publication should follow mutually agreed guidelines (established a priori) and be equitable among partners in high- and low-income settings for the time and efforts contributed and not based on finances/resources contributed.
4.11	Local storage of data are preferred to ensure local and LMIC access to the data. All data/results related to a community should be shared with community leaders prior to publication to prevent any social or emotional harm that can occur with publication of results [60].

### Quality and impact: maximising the relevance, efficiency, accuracy, and reach of collaborative research

Collaborative research in global surgery should aim to maximise the relevance, efficiency, accuracy, and reach of the research being conducted. Summarising the existing literature, we have drafted 13 statements in service of this aim (**Table 6**). Maximising relevance begins with framing and addressing research questions that are relevant to the community or communities in which the research is being conducted [66]. Failure to do so would risk using research participants as experimental subjects without any potential benefit to the individuals or communities being researched. Collaborators who live and work in the communities where the research is being conducted will have a more nuanced and full understanding of the needs and priorities of the community. Rather than imposing research questions and priorities, questions should be developed jointly, involving all collaborators early in the process [70,71]. Clear, timely, frequent, and transparent communication between collaborators facilitates both the development of appropriate questions and the success of the project going forward. This entails making decisions jointly whenever possible as the process moves forward.

**Table 6:** Statements on the theme of quality and impact

5	Quality and impact: maximizing the relevance, efficiency, accuracy, and reach of collaborative research
5.1	Collaborative research work in global surgery should address questions that are relevant to the community or communities in which the research is conducted [66-69].
5.2	Research questions should be developed jointly, involving all collaborators early in the process [68,70-72].
5.3	Communication between all collaborators in the research project should be clear, timely, frequent, and transparent [67].
5.4	Collaborative research in global surgery should emphasize joint decision making between all partners throughout the entire research process [67].
5.5	Clinician-researchers engaging in collaborative research should have protected time to devote to research endeavors [73,74].
5.6	Collaborative research projects in global surgery should be designed and implemented with careful regard for local context and resources.
5.7	An inability to achieve ideal research conditions should not be considered a contraindication to the conduct of collaborative research.
5.8	Partners should strive for the highest level of scientific rigor in study design and analysis while balancing resource constraints in a context specific manner.
5.9	Knowledge generated through collaborative research in global surgery should be widely shared within the community in which the research took place and disseminated through routes accessible to the local population.
5.10	Collaborative research in global surgery should be published in open access journals.
5.11	Knowledge generated through collaborative research in global surgery should be shared at appropriate scientific fora.
5.12	Partnerships should use established assessment tools to regularly assess partnership vision, effectiveness, impact, and design.
5.13	Partnerships should regularly be evaluated based on local impact, cultural appropriateness, and commitment to workforce development.

Often, partners from high-resource settings will have dedicated research time built into their work schedule and salary, whereas those in low-resource settings do not have these protections. Additionally, the clinical burden in low-resource settings can put undue strain on the clinician-researcher [71,73]. Clinician-researchers engaging in collaborative research should have protected time to devote to research endeavours commensurate with their degree of involvement in the project and equitable across sites [74]. Similarly, the cultural and resource context can be vastly different depending on where research is conducted. Collaborative research projects in global surgery should be designed and implemented with careful regard for local context and resources. An inability to achieve ideal research conditions should be viewed as a reflection of the resource setting, not as a contraindication to the conduction of collaborative research. That said, collaborators should always strive for the highest level of scientific rigor in study design and analysis that can be achieved in the given context.

Just as research questions should reflect the needs and priorities of the communities being studied, knowledge generated through collaborative research must be shared via routes that are accessible to the local population. This falls outside the traditional focus of academic medicine, and requires innovative and creative methods of disseminating results. This might entail meetings with community leaders, distribution of results in public spaces, or incorporation of practice and results into local health care settings through collaboration with local providers [70]. Publications generated from collaborative research should reach practitioners who are treating the population in question. One way to achieve this aim is by publishing in open access journals. Similarly, presentations at local, regional, or national conferences should be prioritised.

### Planning for the long term, building capacity, and transferring skills

In equity-oriented partnerships, several areas must be addressed to ensure long-term impact: leadership development, financial stability, and building research capacity. The critical importance of this aspect of collaborative research culminated in 15 statements being drafted (**Table 7**). Development and maintenance of trust in the partnership must be of high priority and is best built through co-creation, shared responsibility, continuous follow up and visible efforts to prioritise LMIC interests. Leadership development involves a commitment to investing in mentorship and faculty development as well as education of trainees. Partnerships should emphasise trainee well-being, equity, and competency-based training. Access to collaborative resources (such as the technology necessary for communication and knowledge sharing) and leadership-focused resources (skills development, mentorship, or coaching) should be made available to participants. Leadership selection and project management should be led by LMIC stakeholders [79,80].

**Table 7:** Statements on the theme of planning for the long term

6	Planning for the long term, building capacity, and transferring skills
6.1	Partnerships should acknowledge the shared need for ongoing ‘value generation’ [32]. Value in partnerships can include professional development, scholarly merit, infrastructure development, clinical productivity, monetary revenue, or building capacity in other ways.
6.2	All collaborators should develop criteria for the success of the partnership, and define parameters for exiting the partnership [7,75].
6.3	Collaborators should aim to build infrastructure to manage funds housed within the institutions and countries in which the research is being conducted [48].
6.4	Long-term funding to achieve stability for the partnership should be sought as early as possible [76].
6.5	Partnerships should commit to investing in mentorship and faculty development. Leadership-focused resources (development, mentorship, coaching) should be made available to participants [74].
6.6	Partnerships should emphasize trainee well-being, equity, and competency-based training.
6.7	Board composition of the partnership should ensure that stakeholders from both sides are not only represented but also empowered to properly represent their communities [76,77].
6.8	The partnership should provide communication resources to participants (for connecting, collaborating, knowledge sharing).
6.9	The partnership should provide mediation resources to participants to manage conflicts and demonstrate commitment to the relationship.
6.10	Opportunities for LMIC partners to lead research (and have first or senior authorship) should be prioritized over opportunities to simply participate [51].
6.11	Development and maintenance of trust in partnership must be of high priority and is best built through co-creation, shared responsibility, continuous follow up and visible efforts to prioritize LMIC interests [3,78].
6.12	Leadership selection and project management should be led by LMIC stakeholders [46,79,80].
6.13	Authorship opportunities for LMIC partners should be targeted with distinct guidance, mentorship, and facilitation from ideation to publication.
6.14	Authorships, budgets, and promotional opportunities should be visibly and equitably shared [81].
6.15	Ownership of physical and intellectual property should be primarily held by LMIC partners [47,61].

Long-term funding to achieve stability for the partnership should be sought as early as possible [82]. Collaborators should aim to build infrastructure to manage funds housed within the institutions and countries in which the research is being conducted.

Research requires attention to shared input from the inception of the idea through ownership of the data and equitable first and senior authorship on resulting presentations and manuscripts; therefore, authorships, budgets, and promotional opportunities should be visibly and equitably shared [81]. Ownership of physical and intellectual property should be primarily held by LMIC partners [47,61]. Because any research pursued in these collaborations should demonstrate value to the community wherein the research takes place, board composition of the partnership should ensure that stakeholders from both sides are not only represented but also empowered to properly represent their communities [76].

Partnerships should acknowledge the shared need for ongoing value generation [32]. Value in partnerships can include professional development, scholarly merit, infrastructure development, clinical productivity, monetary revenue, or building capacity in other ways. The partnership should provide mediation resources to participants to manage conflicts and demonstrate commitment to the relationship. The ultimate goal of any capacity-building enterprise is that each partner will have the capacity to move forward independently. All collaborators should develop criteria for the success of the partnership and define parameters for exiting the partnership [7,75].

## DISCUSSION

To bridge knowledge gaps and improve surgical care worldwide, consensus is required on best practices for collaborative research. As a starting point for a global Delphi process, we assembled an authorship group from a diversity of geographical origins, levels of training, and areas of expertise, conducted a review of the literature across six themes, and drafted a list of 81 equity-focused statements. These statements address salient aspects of partnership building from inception to long-term planning.

We recognise that despite our efforts to minimise bias in the conduct of this work, the questions we chose to ask, our search methodology, interpretations of the literature, discussions, and resulting statements are influenced by the particular perspectives of our authorship group and the power structures that are inherent in collaborative work.

Therefore, to further refine these statements into a practicable guideline, we plan to recruit a Delphi panel consisting of a broad range of individuals who are representative of the spectrum of parties involved in global surgery collaborations: physicians, non-physician researchers, nursing and other allied health leaders, research funders, surgical college leadership, non-governmental organisations, and industry. We will recruit from all world regions. We plan to conduct four survey rounds, beginning with a qualitative round during which individuals can provide feedback on the initial 81 statements and suggest others. During the subsequent quantitative rounds, statements will be ranked on a nine-point Likert scale ranging from ‘strongly disagree = 1’ to ‘strongly agree = 9’ [83,84]. If over 80% of respondents rate a statement 7–9 (moderately through strongly agree), or 1–3 (strongly through moderately disagree), consensus will be considered reached and the statement dropped from subsequent rounds. The statements with a consensus ‘agreement’ will be kept in the final guideline, whereas those with a consensus ‘disagreement’ will not. Panel participants who contribute to all rounds of the process will be invited to serve as co-authors on the final guidelines manuscript.

Given the ambitious scope of these guidelines, this Delphi process will add additional real-world perspectives on the challenges that may be faced in implementing these guidelines across a variety of settings. The qualitative portion in particular will allow panelists to provide a ‘reality check’ on whether each represents a reasonable expectation for collaborative teams.

## CONCLUSIONS

Current practice in global surgery research collaborations is highly variable and there is no universal standard by which collaborations can be assessed. By leveraging existing literature and building consensus formally via a Delphi process, we aim to establish standards for impactful and equity-focused collaborations.
